# Feasibility of Osteopathic Manipulative Treatment for Low Back Pain in Rural Chimaltenango, Guatemala: A Pilot Study

**DOI:** 10.7759/cureus.90946

**Published:** 2025-08-25

**Authors:** Monica Aspra Rubi, Ambrose Loc T Ngo, Grace Cooper, Jared Nichols

**Affiliations:** 1 College of Osteopathic Medicine, Kansas City University, Joplin, USA; 2 Osteopathic Medicine, Kansas City University, Joplin, USA

**Keywords:** global health, guatemalan healthcare, low back pain, osteopathic manipulation treatment, rural healthcare

## Abstract

Background

Low back pain (LBP) is a global health issue that primarily affects underserved communities with limited access to healthcare. Osteopathic manipulative treatment (OMT) offers non-pharmacological, hands-on interventions that align with its core principle of holistic care. However, little is known about the feasibility and perception of OMT in rural Chimaltenango, Guatemala. This pilot study assesses the feasibility, effectiveness, and participant perception of osteopathic principles in managing LBP complaints.

Methods

Adult participants experiencing LBP were recruited by the research team during Kansas City University College of Osteopathic Medicine’s (KCUCOM) global health mission trip, following their one-day primary care visit. Pre- and post-treatment Visual Analog Scale scores (VAS), Oswestry Disability Questionnaire (ODQ), and modified Osteopathic Survey of Health Care in America (OSTEOSURV) surveys were used in the study to assess pain, activities of daily living, satisfaction, and awareness of OMT. Manipulative techniques were tailored based on the participants' complaints and delivered by a trained fourth-year medical student under the supervision of an osteopathic physician.

Results

The data showed that 62.5% (n = 5/8) of participants reported a baseline pain score of VAS ≥ 8. Post-treatment, the majority, 75% (n = 6/8), reported greater than 50% pain relief, and 12.5% (n = 1/8) were pain-free (VAS = 0). All participants (100%, n = 8/8) were unfamiliar with OMT before the session. However, 75% (n = 6/8) reported satisfaction with the treatment following manipulation.

Conclusion

OMT is a feasible and acceptable modality for LBP management in rural Guatemala. These findings support further investigation that includes a larger sample size and the possibility for future integration of osteopathic care into global health outreach, particularly for under-resourced populations that lack access to conventional pain management.

## Introduction

Low back pain (LBP) is the leading cause of disability worldwide, and the condition for which non-surgical interventions may be of greatest help. In 2020, approximately 619 million people experienced LBP globally, with cases expected to rise to 843 million by 2050 [1]. In Guatemala specifically, the prevalence of LBP is estimated to be 14.8% as reported in a national survey [2]. The guidelines for caring for individuals experiencing LBP should be holistic and equitable, highlighting socioeconomic factors that may influence the diagnosis [1]. Additionally, rural and indigenous populations in Guatemala are the least likely to receive adequate care due to a lack of access to care, dialect language barriers, mistrust in providers, and their reliance on local traditional healers [3]. Further compounding this disparity, the World Health Organization recommends a minimum of 44.5 healthcare workers per 10,000 people to achieve adequate health coverage; however, rural regions in Guatemala often have as few as 12.5 per 10,000 people, highlighting the critical shortage these communities experience [4]. Cultural sensitivity and trust are essential when providing osteopathic manipulative treatment (OMT) for LBP complaints. The Kansas City University College of Osteopathic Medicine (KCUCOM) has been conducting medical trips to rural parts of Guatemala for over 20 years, where students of osteopathic medicine have the opportunity to engage with the community and promote osteopathic principles that are not commonly known by Guatemalans, since there are no formal osteopathic programs within the country. There are only two DOCARE clinics in Guatemala, and as an organization, they host short-term medical trips [5].

Given this context, our analysis evaluated whether patients at different clinics in Chimaltenango, Guatemala, hold a positive perception of osteopathic medicine, particularly in the context of KCUCOM's 20-year history in providing medical care to the region. Based on qualitative and quantitative findings from eight participants, we aim to highlight the value of incorporating alternative therapies to treat LBP. This pilot study aims to study the feasibility of implementing OMT and integration of osteopathic care with traditional practices and community education to treat LBP in underserved populations.

## Materials and methods

This pilot study aimed to assess the feasibility of implementing OMT in a rural Guatemalan clinic. It involved a targeted review of existing literature, including systematic reviews, randomized controlled trials, and meta-analyses, to guide its design and methodology. Participants were eligible for inclusion if they were over the age of 16, had the cognitive ability to provide informed consent, were able to complete the Oswestry Disability Questionnaire (ODQ), the Osteopathic Survey of Health Care in America (OSTEOSURV), and Visual Analog Scale (VAS) surveys, and self-identified as experiencing LBP for greater than six weeks [6-8]. Of note, all surveys were translated into Spanish, and the OSTEOSURV-I used was shortened for this study. Individuals were excluded if they were under the age of 16, lacked the cognitive ability to provide informed consent, presented with musculoskeletal complaints unrelated to LBP, or had comorbid conditions that could confound OMT, such as severe osteoporosis [9].

Participants were identified for study participation following their primary care visit by informing the researcher that they were potential participants for the study. The researcher would then inform the patient about the study, determine if they met the criteria, and ask for their consent to participate. Once participants agreed to participate in the study, following informed consent, they completed the ODQ and VAS before receiving treatment. The researcher then provided a patient-specific OMT to offer LBP relief. The osteopathic manipulative medicine (OMM) modalities used for each patient were recorded on a sheet that had their associated patient number, who performed said technique, and how long the treatment was given. This was all manually recorded on an OMM technique spreadsheet. Once participants received treatment, they completed the modified OSTEOSURV and post-treatment VAS.

Intervention

All participants received OMT customized to their specific needs and clinical presentations. The treatment approach emphasized patient-centered care and was adjusted based on subjective reports and somatic dysfunctions found during physical examination. These included myofascial release, soft tissue techniques, counterstrain, and muscle energy techniques. Studies have compared the efficacy of different OMT procedures in treating LBP, and the results have shown that myofascial release provides the most benefit to patients [10]. Even though OMT is highly efficacious in treating musculoskeletal complaints, it remains underutilized in many healthcare settings [11].

Outcomes

**Figure 1:**
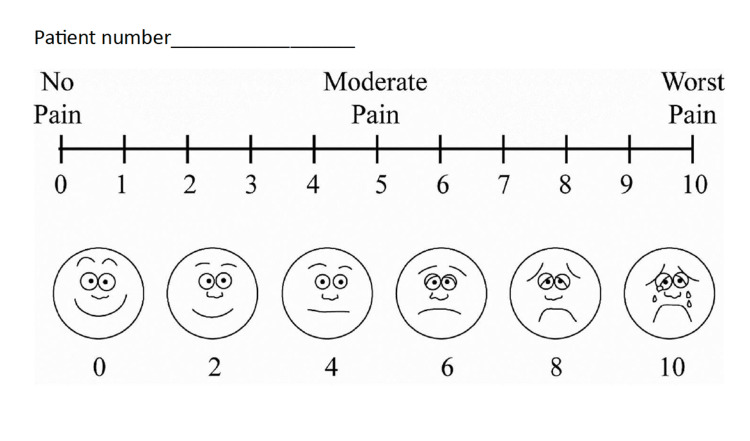
Visual Analog Scale (VAS) score Credit: Reference [8]

The study assessed several outcome measurements to evaluate the participants' perceptions and satisfaction with OMT. Pain management was measured using pre- and post-treatment VAS (Figure 1), developed by M.H.S. Hayes and D.G. Patterson [8]. Overall satisfaction with the intervention was measured using a modified OSTEOSURV survey, developed by the Journal of Osteopathic Medicine and the American Osteopathic Association [7]. Participants’ understanding of osteopathic principles was evaluated through post-treatment qualitative responses and survey data.

Study design and analysis

The pilot study is a pre-post analysis designed to explore the practicality, acceptability, and perceived effectiveness of OMT for LBP within the rural Guatemalan population. The study utilized quantitative data (VAS scores and ODQ) and qualitative observations (patient satisfaction and perception of osteopathic care). Data collection involved structured surveys and patient-reported outcomes. Additionally, team members utilized descriptive statistics, such as Wilson's score interval for binomial proportions, p-values calculated using Fisher’s exact test due to the small sample size, chi-square test to compare more than two categories, and confidence intervals (CI) using Microsoft Excel (Microsoft® Corp., Redmond, WA, USA).

Recruitment of participants

During the study period, the practitioner provided primary care to approximately 5-10 patients daily over three and a half days. Of the 12 eligible patients invited to participate, eight consented and were enrolled. The reasons for declining participation were not formally recorded; however, informal comments suggested possible factors such as time constraints, lack of interest in research participation, or unfamiliarity with OMT. Once enrolled, no participants declined to receive OMT. Informed consent was obtained prior to enrollment, and all eligible patients were provided with an informational sheet or assent form for review.

Initial survey administration

Participants who provided consent were separated from other patients to ensure privacy during data collection. They then completed the ODQ developed by Jeremy Fairbank and Graham Pynsent and the VAS scores (Figure 1) [6]. For participants with limited literacy, a trained researcher administered the surveys verbally, reading directly from a standardized script in either English or Spanish. The researcher received training in medical Spanish through the completion of a certified course offered by Canopy. Demographic data, including age range and gender, were collected alongside questions about whether they have reliable access to pain relief medication and their past surgical history. To maintain anonymity, each participant was assigned a unique, randomly generated identification number, and all completed surveys were stored securely and de-identified.

Pre- and post-treatment OMM survey

Following the initial survey, each participant received OMT for their lower back pain. The practitioner documented all treatments on a standardized OMT technique log. Each patient received at least three OMT treatments that were specific to their complaint and findings while performing an osteopathic screen. Upon completion of a one-time intervention, participants completed the modified OSTEOSURV survey and a post-treatment VAS score (Figure 1) [8]. The OSTEOSURV and VAS scores were available in Spanish, and the same translation protocol was used as described above.

Participant safety and follow-up

To minimize potential risk, a licensed osteopathic physician remained on-site during the study to provide immediate support. This included local physicians affiliated with DOCARE clinics and the Hermano Pedro Association, which is a community program that provides medical treatment, offers follow-up care, and support for participants requiring further care.

Ethical considerations

This study was reviewed and approved by the Institutional Review Board (IRB) at KCUCOM under protocol number 2056610-2. All participants provided informed consent before participation, with verbal explanations provided in Spanish as needed. Special accommodations were made for participants with low literacy. All data were anonymized and securely stored in accordance with the ethical standards for research involving human subjects.

## Results

Pain level reduction

**Figure 2:**
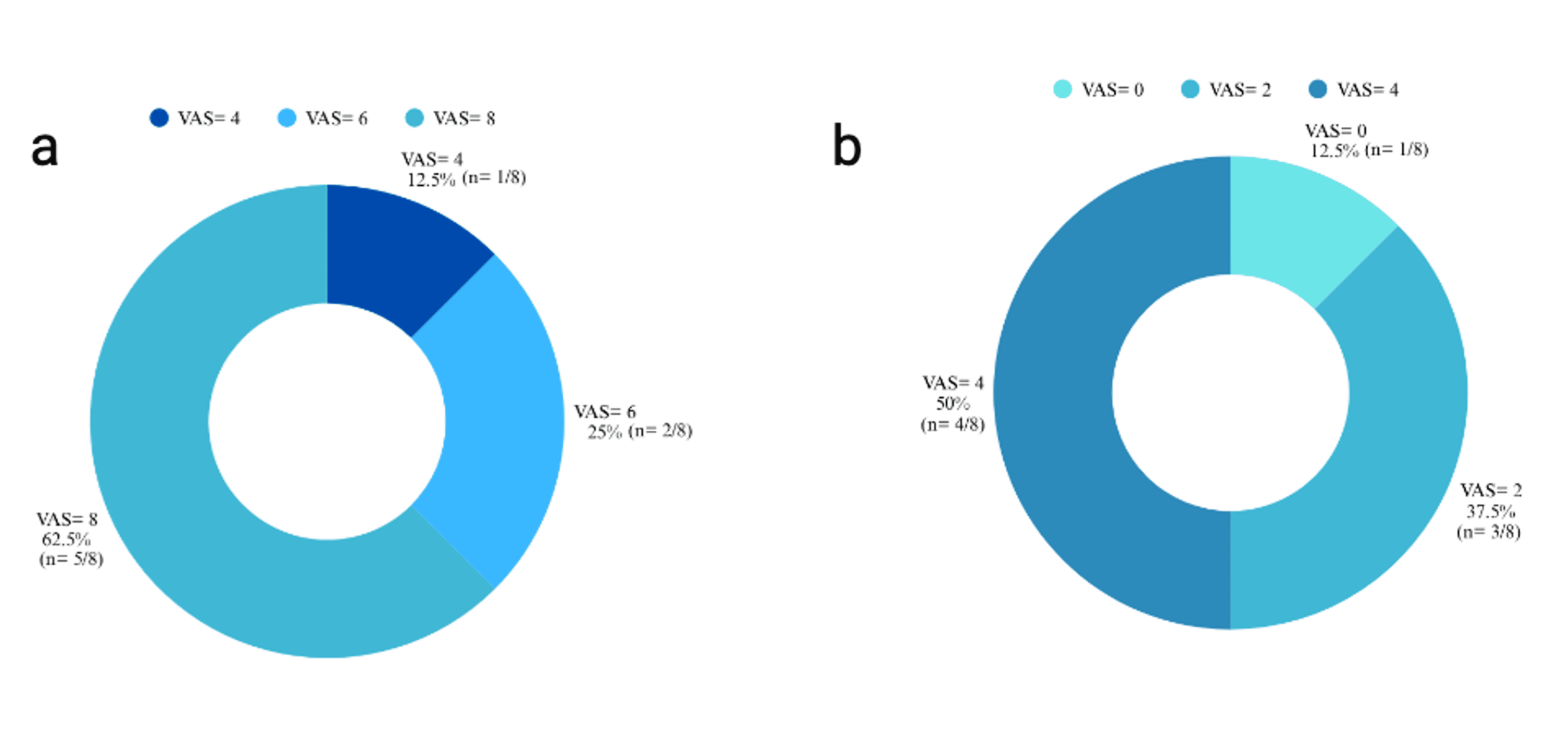
(a) Mean scores for pre-OMT VAS; (b) Mean scores for post-OMT VAS. (a) VAS scores ranged from 0 to 10. Donut chart representing VAS pain scores before osteopathic manipulative treatment (OMT) among eight participants. Confidence intervals (CIs) were calculated using the Wilson method (95% CI: 31%-86%). Statistical significance was considered at p < 0.05. (b) VAS scores ranged from 0 to 10. Donut chart showing VAS pain scores following one session of OMT among eight participants. Data are presented as percentages (%, n). CIs were calculated using the Wilson method. Statistical significance was considered at p < 0.05. VAS: Visual Analog Scale

Before treatment, 62.5% (n = 5/8) of patients had a pain score of VAS = 8, followed by 25% (n = 2/8) with VAS = 6, and 12.5% (n = 1/8) with VAS = 4 (Figure 2). This distribution reflects the high baseline discomfort reported by most participants. After a single OMT session, pain scores decreased across participants, with 50% (n = 4/8) reporting a VAS score of 4, 37.5% (n = 3/8) reporting a VAS score of 2, and 12.5% (n = 1/8) reporting complete pain relief (VAS = 0) (Figure 2).

Following treatment, 75% (n = 6/8; 95% CI: 40%-93%) of patients reported at least 50% or more relief, and 12.5% (n = 1/8; 95% CI: 2%-51%) achieved complete pain relief (VAS = 0). Nonetheless, some patients continued experiencing pain, albeit at a reduced level compared to their baseline.

Pre-OMT functional impacts

Among the study participants (N = 8), 75% (n = 6/8; 95% CI: 40%-93%) reported difficulty with personal care tasks such as dressing, grooming, or lifting heavy objects due to pain. Approximately 87.5% (n = 7/8; 95% CI: 53%-98%) experienced pain specifically when lifting objects. Additionally, pain prevented 33% (n = 2/8; 95% CI: 12%-65%) of participants from walking more than a mile, 50% (n = 4/8; 95% CI: 24%-76%) from standing for more than an hour, and 62.5% (n = 5/8; 95% CI: 31%-86%) from sitting in a chair for more than an hour.

Furthermore, 75% (n = 6/8; 95% CI: 40%-93%) reported that traveling causes pain, and an equal proportion reported requiring medication to sleep. Despite this, 25% (n = 2/8; 95% CI: 7%-60%) of participants reported sleeping fewer than six hours even with medication. Notably, 87.5% (n = 7/8; 95% CI: 53%-98%) stated that pain does not affect their social life. Table 1 highlights the aforementioned results for the study’s eight participants.

**Table 1:** Mean scores of functional impact from low back pain (LBP) among participants pre-OMT (n = 8). Data are presented as n/N, percentages (%), and p-values calculated using Fisher’s exact test due to the small sample size. Each response was compared to an expected 50% distribution under the null hypothesis. Statistical significance was considered at p < 0.05. OMT: osteopathic manipulative treatment

Functional Impact	Participants (n/N)	Percentage (%)	P-value (Fisher’s Exact)
Difficulty with personal care	6/8	75	0.608
Pain from lifting objects	7/8	88	0.282
Pain preventing walking >1 mile	4/8	50	1.000
Pain preventing standing >1 hour	4/8	50	1.000
Pain preventing sitting >1 hour	5/8	63	1.00
Pain with traveling	6/8	75	0.608
Pain relief medication needed for sleep	6/8	75	0.608
Sleep <6 hours with medication	2/8	25	0.608
Social life not impacted by pain	7/8	88	0.282

Access to healthcare and medical awareness

Limited healthcare access was evident, as no patients (n = 0/8, 0%) had health insurance, and 38% (n = 3/8; 95% CI: 15%-70%) did not see a physician in the past year. Furthermore, there was a complete absence of knowledge about the field of osteopathic medicine, as all patients (n = 8/8, 100%) were unsure of what osteopathic treatment involves.

Demographic trends

The patient population was primarily composed of Hispanic and Indigenous Mayan individuals, with 38% (95% CI: 15%-70%) being Hispanic and 62% (95% CI: 30%-85%) being Indigenous Mayan. A total of 62% (95% CI: 30%-85%) reported that they had not completed high school. Their employment also indicates socioeconomic strain, as 13% (95% CI: 2%-51%) of them were unemployed, and 62% (95% CI: 30%-85%) were stay-at-home homemakers (Table 2).

**Table 2:** Mean scores for participant demographics (n = 8). Data are presented as n/N, percentage (%), chi-square test statistic (χ²), and p-values. A chi-square goodness-of-fit test was used to assess whether observed frequencies differed significantly from equal distribution. Statistical significance was considered at p < 0.05. OMT: osteopathic manipulative treatment

Variable	Participants (n/N)	Percentage (%)	Chi-Square Test (χ²)	P-value
Sex (female vs. male)	5/8 vs. 3/8	62.5 vs. 37.5	0.50	0.480
Age range (16-29, 30-49, 50+)	2/8 vs. 4/8 vs. 2/8	25 vs. 50 vs. 25	1.00	0.607
Ethnicity (Indigenous Mayan vs. Hispanic)	5/8 vs. 3/8	62.5 vs. 37.5	0.50	0.480
Education level (did not complete high school vs. did complete high school)	5/8 vs. 3/8	62.5 vs. 37.5	0.50	0.480
Employment status (homemaker vs. employed vs. unemployed)	5/8 vs. 2/8 vs. 1/8	62.5 vs. 25 vs. 12.5	3.25	0.197
Did not have access to a physician in the past year vs. did have access to a physician this past year	⅜ vs. 5/8	37.5 vs. 62.5	0.50	0.480
No health insurance coverage	8/8	100	-	-
No previous knowledge of OMT	Unfamiliar	8/8	-	-

Patient satisfaction and perceptions

The views of patients toward osteopathic treatment varied. Six out of eight participants (n = 6/8, 75%; 95% CI: 40%-93%) reported satisfaction with the treatment, while two participants (n = 2/8, 25%; 95% CI: 7%-60%) were neutral or reported only slight to moderate improvement. Although participants noted varying degrees of improvement, there was no strong opposition to osteopathic treatment, and all expressed a willingness to explore different strategies for pain relief.

## Discussion

Based on the findings from this feasibility study, implementing OMT for LBP appears to be a practical and acceptable approach in rural settings such as Chimaltenango, Guatemala. However, a larger sample size is needed to state this, and this pilot aims to get a better understanding of whether OMT can be an alternate therapy for populations that are under-resourced. Despite the patients' unfamiliarity with OMT, we concluded that it decreased immediate pain in the eight participants involved, and patients reported that OMT could improve their quality of life, and six out of eight patients reported positive perceptions post-OMT. One of the key achievements of this study was the improvement in pain levels among patients who received OMT treatment. With the baseline measurements, participants experienced at least moderate to severe pain (VAS 6-8), with a fraction of participants enduring debilitating pain (VAS ≥ 8). Subsequently, their post-treatment assessment showed that several people mentioned that their pain dropped by half or more, with some feeling immediate relief. The documented results align with the existing literature on the efficacy of OMT in treating musculoskeletal disorders. As noted, even a single treatment or short-term intervention can provide pain relief benefits [12,13]. It is of key importance to note that at this time, generalizable results cannot be made as a larger sample size and a longitudinal study are needed.

In addition to reported pain reduction, participants experienced improvements in mobility and the performance of daily tasks. The eight participants reported pain experienced during their daily activities (e.g., walking, lifting objects, personal care) pre-OMT; however, post-OMT, six out of eight participants agreed that OMT may have the potential to reduce LBP, and had a positive perception of osteopathic philosophies. The overall satisfaction finding is important in low-resource settings where access to medication is often limited. However, the integration of OMT in rural Guatemala encounters inadequate healthcare infrastructure, limited insurance coverage, and a general lack of public awareness about osteopathic medicine. Most participants reported having no health insurance and infrequent contact with healthcare providers, which highlights gaps in routine and preventive care. Despite the fact that KCU-affiliated teams have engaged with the Chimaltenango region for approximately 20 years, participants in this pilot study reported little to no prior familiarity with OMT. This apparent discrepancy may reflect a combination of factors, including the lack of sustained or standardized community education regarding OMT, potential terminology and health literacy gaps, cultural interpretations of hands-on musculoskeletal care, and variability in outreach personnel over time. Previous engagements may have prioritized episodic clinical care over longitudinal awareness-building, leading to fragmented recognition of the modality. Recognizing this, the current study included an introductory educational component about OMT to ensure informed participation. Despite these difficulties, post-treatment comments showed a positive attitude towards OMT, and most patients depicted great treatment satisfaction and a willingness to use the treatment in the future if it were accessible.

Additionally, five out of the eight participants have less than a high school education and are either unemployed or stay-at-home, indicating possible socioeconomic barriers that may be present at large in Chimaltenango. Most individuals engage in physically demanding work, making musculoskeletal pain highly prevalent. The lack of access to basic medical care further compels reliance on self-care. Results indicate, alongside other limitations, that OMT is an inexpensive solution, offering a non-invasive treatment alternative particularly suitable for severely economically and geographically constrained populations.

The southern regions of Guatemala show potential for utilizing OMT treatment for LBP. The need for increased healthcare access and encouraging osteopathic exposure has the possibility to increase perceptions and disseminate alternative treatments to the community as a whole. Future work may focus on optimizing informational resources on basic osteopathic principles and enabling OMT to become a practical, hands-on option for local healthcare workers in helping patients manage muscle and joint pain.

Limitations

There are key limitations in this pilot feasibility study that must be kept in mind while interpreting the results. To begin with, the findings of this study are not intended to assess clinical efficacy, but rather to assess logistical, cultural, and operational feasibility, which is a crucial first step before any future large-scale or outcomes-based trials can be appropriately designed and conducted in similar contexts. Next, the small sample size in this study (n = 8) significantly restricts the statistical power and generalizability. The study did not include a control group, as the study was not designed or powered to assess the clinical effectiveness of OMT or include a control group. Furthermore, while OMT was provided as the patients were seen in the clinic, the documentation of the techniques used was general in nature, which poses concerns related to reproducibility. The clinical setting’s time constraints and short duration directly impacted participant recruitment and data collection. There was variability in recruitment across clinic days due to time constraints, which may contribute to selection bias.

In addition to limitations of the study design, there are other contextual factors that affect the feasibility of delivering OMT in this rural Guatemalan context. Socio-economic and educational barriers, such as low levels of literacy, limited healthcare access, and diversity in language and culture, also play a role. Trust barriers established between healthcare providers and indigenous communities also shaped how participants engaged with the study. Though these factors do not serve as design limitations of the study, they are essential to understanding the feasibility challenges and should be considered while planning extensive research in comparable contexts.

Future directions

As a pilot project, several unforeseen challenges emerged, many of which can be addressed in future iterations, since this trip is conducted annually. Based on the flow of clinic days, designating specific time blocks (such as a dedicated half-day) for research activities could improve both time management and increase sample size. Additionally, having more than one researcher available to enroll participants could reduce workflow interruptions and streamline data collection. The generalizability of this study is limited by the small sample size (n = 8). Implementing these changes in future studies may enhance the development of this project and contribute to a better understanding of OMT for LBP.

Gaining a deeper understanding of cultural traditions can aid future researchers in building trust with their participants. By taking the time to familiarize themselves with local colloquialisms and basic Mayan dialects, researchers can break down communication barriers, leading to more accurate data collection and stronger engagement from participants.

Moreover, incorporating an educational aspect into future interventions could tackle some ongoing healthcare challenges that these resource-limited areas face. For example, teaching local doctors, nurses, and community health workers simple osteopathic techniques could enhance the study's impact, fostering both immediate and lasting improvements in community health. However, to ensure effectiveness and avoid misapplication, such training would need to include sufficient supervised practice and assessment of clinical proficiency prior to independent implementation. With more data gathering in future projects, more changes can be made to help these communities. Overall, future studies with an expanded scope and rigorous design to build upon these preliminary findings are needed.

## Conclusions

This pilot study highlights the feasibility and potential value of OMT in addressing LBP among underserved populations in rural Guatemala. Although participants were unfamiliar with osteopathic care, they reported notable improvements following a single treatment, including reduced pain, increased mobility, and overall satisfaction after one treatment session. These results are consistent with existing literature and support that OMT may serve as a viable, low-risk alternative to pharmacologic approaches, particularly in areas with limited access to conventional medical care. Nevertheless, the positive participant response, combined with OMT’s low-risk and non-pharmacologic nature, suggests potential for integration into existing local healthcare frameworks. Future research with larger samples and longer follow-up is needed to evaluate sustained effectiveness and scalability. Overall, these findings support further exploration of OMT as a sustainable, patient-centered option for reducing musculoskeletal pain in marginalized rural populations.
